# Editorial: Next-generation innovation policies: Promoting systemic socio-economic transformative change

**DOI:** 10.3389/frma.2023.1146039

**Published:** 2023-02-13

**Authors:** Marina Ranga, So Young Kim

**Affiliations:** ^1^Faculty of Management, University of Warsaw, Warsaw, Poland; ^2^Korea Policy Center for the Fourth Industrial Revolution, Korea Advanced Institute of Science and Technology (KAIST), Daejeon, Republic of Korea

**Keywords:** transformative innovation policy, sustainable transitions, systemic innovation, Smart Specialization, emerging technologies (AI, blockchain), Recovery and Resilience Plan, transformative government

In response to complex contemporary challenges, innovation policies of recent years have adopted new orientations that mark a transition from the dominant focus on technological, organizational, and marketing innovations of the so-called “second frame of innovation policies” (Schot and Steinmueller, 2018), focused on competitiveness and economic growth by stimulating learning and enabling entrepreneurship, toward a “third frame” that also considers persistent environmental and social challenges and the need for sustainable transitions and transformative change (e.g., Geels, 2002, 2004; Kivimaa and Kern, 2016; Fagerberg, 2018; Tödtling and Trippl, 2018; Diercks et al., 2019; Coenen and Morgan, 2020; Ghosh et al., 2021; Haddad et al., 2022).

This implies a shift from single-sector policy objectives, such as improving the overall functioning of the research and innovation system, to a multi-sector, systemic perspective that acknowledges the economic, technological and societal determinants of innovation and requires an integrated outlook. Such system-level transformative change requires linkages across multiple stakeholders and policy domains, identifies and exploits synergies, and designs new policy and regulatory instruments to improve coordination, priority-setting and resource allocations. It entails deep changes in a country or region's institutional setups and institutional capacity: it seeks to increase availability of specialized human capital and industrial infrastructures, to improve the capacity to generate and absorb new technologies, to design new production and consumption patterns, and improve the quality of the environment and living standards (transportation, health, food supply, housing, etc.).

Innovation has become more tightly connected with the grand societal challenges addressed by the UN Sustainable Development Goals (SDGs) (e.g., Coenen et al., 2015; Tödtling et al., 2022; Voegtlin et al., 2022). At EU level, concepts such as responsible research and innovation (RRI) (Stilgoe, Owen, and Macnaghten, 2013; Von Schomberg, 2013; Thapa et al., 2019) and mission-oriented research and innovation reflect this new focus on societal goals, inclusion and sustainable transitions (Mazzucato, 2018; Hekkert et al., 2020; Hill, 2022).

Smart Specialization Strategies (S3), a concept that has been at the heart of the European Commission's Cohesion Policy, has also contributed to the development of next-generation innovation policies through its place-based approach and regional innovation objectives (Foray, 2018; Asheim, 2019; Trippl et al., 2020). Introducing the “entrepreneurial discovery process” as a bottom-up, collective involvement of innovation stakeholders from several socio-economic sectors that work together to define and realize national and/or regional priorities, S3 shed new light on the intricate connections between innovation and other sectors, especially industry, education and regional development. Recent studies on the experience of implementing S3 in countries and regions across the EU document both the strengths and weaknesses of the approach. On the one hand, positive impacts have been identified, such as a more methodical planning, higher quality and effectiveness of coordination and collaboration between actors, making the governance of innovation policy in the regions more inclusive, as well as higher stakeholder involvement and trust between private and public actors (Marinelli and Perianez Forte, 2017; Guzzo and Perianez Forte, 2019; Guzzo and Gianelle, 2021). New ways in which S3 can be used to address sustainability challenges and the SDGs have also been proposed (Miedzinski et al., 2021). On the other hand, S3 implementation has also identified many weaknesses that require further attention, especially in the less developed countries and regions. Among them are the risk of greater polarization arising from low institutional capacity to effectively mobilize funds (Incaltarau et al., 2020), gaps in government coordination and policy-making capacity (Guzzo and Gianelle, 2021), a narrow understanding of innovation/S3 based on R&D and knowledge-intensive firms (Hassink and Gong, 2019), protection and subsidies for existing industries regardless of their potential and dynamism (Fedeli et al., 2019), and a stronger focus on supporting niche innovations rather than introducing mechanisms for scaling up and generating a transformative impact (Miedzinski et al., 2021).

Innovation governance has been explored through new interdisciplinary research at the junction between innovation policy and public policy, identifying new roles of government in policymaking and policy implementation and introducing new concepts or revisiting existing ones in light of the new challenges. Some examples include the concept of transformative governance, in the sense of novel roles of public administration that extend beyond the public sector boundaries, better government capacity to address societal and environmental challenges and better use of policy intelligence to develop alternative future scenarios and transition pathways, more resilient to disruptions (e.g., Bos and Brown, 2014; Borrás and Edler, 2020; Braams et al., 2021). Also included here are the whole-of-government approach that aims to improve collaboration between government departments and levels (Christensen and Lægreid, 2007) and the multi-level governance perspective that seeks to improve the intricate connections between regional, national and EU levels. Governance patterns in centralized vs. decentralized institutional setups and the ways in which authority, legitimacy, trust and power are built and exerted have also been explored in more detail, especially in connection with the effectiveness of S3 implementation. All these studies have pointed out to the fact that new policy design and implementation mechanisms also need new mindsets and institutional cultures that may take significant time and effort to introduce in the broader governance systems in which they operate.

This research collection brings together a set of four original research articles that take an in-depth look at the mechanisms by which next-generation innovation policies are designed and implemented in different socio-economic contexts. This new evidence is aimed to inform and inspire the work of academic researchers, government policy makers and public administrators, as well as other innovation stakeholders involved in the design and adoption of next-generation innovation policies.

In their paper “*Implementing systemic innovation strategies for a more sustainable future: the case of three overseas countries and territories*,” Toffanin and von Gesseneck acknowledge the accelerated economic, social and environmental changes that occurred in recent years and generated new trends and processes that interact unpredictably and with unintended consequences. The authors observe the new focus on sustainability in innovation policies and the need for radical systemic transformations in multiple sectors, including policy, culture, and civil society. They also appreciate the difficulty of the task, due to the lack of prior experience with the governance of critical local and global sustainability issues. Therefore, it is suggested that a policymaking approach seen as a collective learning process and as social endeavor rather than a mechanism for imposing decisions might be more suitable to the new circumstances. Based on a study aimed to promote innovative approaches to development in the EU's Overseas Countries and Territories, the authors propose a shared methodology based on “backcasting,” a specific type of foresight, to facilitate policy learning and policymaking within a wide range of territories, regardless of their wealth, geographic characteristics and internal political organization. This innovative approach adopts a systemic innovation perspective and identifies novel ways for defining policies for sustainable development and long-term transformative change.

Kalenzi's paper “*Artificial intelligence and blockchain: How should emerging technologies be governed?*” examines a key topic for the digital transition: the governance and development of emerging technologies, with a focus on blockchain and artificial intelligence (AI). The paper maps new platforms within public, private and civil society, identifies major players and explores their underlying motivations. The analysis of divergence and convergence in motivations and their impact on the governance of emerging technologies yields very interesting insights. On the one hand, there is a broad consensus among the major players on the role of these technologies as key drivers of current and future economic growth, and on their significant risks to society. On the other hand, there is considerable confusion and disagreement on the ways to achieve a proper balance between technology development and risk mitigation. Responses vary from calls for self-regulation to calls for strong state regulation and monitoring of these technologies. Considering the likelihood of persisting disagreements in the foreseeable future that may affect the optimal development of governance ecosystems across jurisdictions, the author proposes a review of the existing legal and institutional frameworks to protect consumers and society from the risks of these technologies, and as needed, an update or the introduction of new amendments to cover for various novel uses of the new technologies. This approach is thought to facilitate and advance the understanding of governance challenges that these emerging technologies pose to society, as no single government, industry and civil society player has a clear answer on the fine balance between promotion of these technologies and limitation of their risks to users.

The third paper—“*S3 and Recovery & Resilience Funds: a case study built on the experience of ten Spanish regions*”—authored by De Molina et al., examines a critical issue for next-generation innovation policies: policy alignment and coordination. In a study of 10 Spanish regions (autonomous communities), the authors look at the connections between the national Recovery and Resilience Plan designed in the context of the Next Generation EU package, and the existing regional Smart Specialization Strategies (S3) and related ecosystems. They acknowledge key contributions of S3 to the Spanish regions, such as participative governance, creation of regional ecosystems, and a change of vision in public intervention, but also remark an important policy gap: the lack of an ex-ante alignment between the drafting of the national Recovery and Resilience Plan and the experience of the regional S3 strategies and regional S3 ecosystems. This led to a disconnect between the recovery logic (based on national public consultations to identify strategic projects) and the S3 logic (based on a strategic priority-setting exercise conducted by each regional ecosystem) that may weaken the recovery planning process if the multi-actor, multilevel and place-based S3 approach are not considered. An ex-post correction of this misalignment between the two processes is thought to still be possible, to protect existing regional shared visions, and should offer a clear recognition of the S3 ecosystems and S3 managing bodies, as well as the significant role that S3 could play in the recovery process.

Finally, the paper “*Outlining the orientation toward socially relevant issues in competitive R&D funding instruments*” by Spinello et al. examines another critical issue for the next-generation innovation policies: the relevance and orientation of project-based funding in public R&D investments. The authors note that, despite the growing importance of this type of funding in all European countries over the last two decades, the actual orientation of project funding instruments to promote innovation and wellbeing remains a matter of concern for decision-makers. Public R&D investment is thought to have the capacity to improve the citizens' quality of life if they pursue “relevant” existing and/or emerging societal objectives. In the case of project-funded research, “relevance” in research objectives is related to creating “usable results” from public investments. The paper uses recent government R&D funding data collected at European level to examine the portfolio of policy instruments for funding the research projects of various public research funding organizations (RFOs) and to shed light on their social relevance. The authors find that, beyond the declared objectives, there are several factors that influence the actual orientation of funding instruments and these are related to the implementation process operated by the RFOs, such as the beneficiary selection process, the evaluation criteria and their importance, and the composition of evaluation panels.

These four papers address only a handful of the key dilemmas and concerns in devising and implementing next-generation innovation policies, from the multitude and variety of topics and issues that need further exploration for enabling policymaking and governance frameworks to meet the contemporary innovation challenges. In this time of growing complexity and uncertainty, and ever-tighter integration of policy, technology, society and environment, this research collection is a small step toward the grand challenges of understanding and developing next-generation innovation policies that can induce systemic socio-economic transformative change.

## Author contributions

MR proposed the concept and co-authored the editorial. SK co-authored the editorial. All authors contributed to the article and approved the submitted version.
